# Novel Experimental Setup for Coulometric Signal Transduction of Ion-Selective Electrodes

**DOI:** 10.3390/membranes12121221

**Published:** 2022-12-02

**Authors:** Naela Delmo, Zekra Mousavi, Tomasz Sokalski, Johan Bobacka

**Affiliations:** Laboratory of Molecular Science and Engineering, Johan Gadolin Process Chemistry Centre, Åbo Akademi University, Henriksgatan 2, FI-20500 Turku-Åbo, Finland

**Keywords:** coulometric signal transduction, solid-contact ion-selective electrodes, conventional ion-selective electrodes, PEDOT:PSS, MWCNT, chronoamperometry, chronocoulometry, electrochemical impedance spectroscopy

## Abstract

In this work, a novel and versatile experimental setup for coulometric signal transduction of ion-selective electrodes (ISEs) is introduced and studied. It is based on a constant potential coulometric measurement carried out using a one-compartment three-electrode electrochemical cell. In the setup, a potassium ion-selective electrode (K^+^- ISE) is connected as the reference electrode (RE). A poly(3,4-ethylenedioxythiophene) doped with polystyrene sulfonate (PEDOT:PSS)-based electrode with a dummy membrane (DM) and a glassy carbon (GC) rod are connected as the working electrode (WE) and counter electrode (CE), respectively. Adding a non-selective dummy membrane to the structure of the WE facilitates the regulation of the measured signal and response time. The results from electrochemical impedance spectroscopy measurements carried out on the WE showed that the time constant is profoundly influenced by the dummy membrane thickness. In addition, the redox capacitance of the PEDOT:PSS film shows a better correlation with the electrode area than the film thickness. Sequential addition/dilution experiments showed the improvement of current and cumulated charge signals in the new setup studied in this work compared to the setup used in the original coulometric signal transduction method. Both conventional ISEs and solid-contact ISEs (SCISEs) were used in this work. The results showed that the coulometric response was independent of the type of ISE used as RE, confirming the versatility of the novel set-up.

## 1. Introduction

In environmental monitoring and clinical point-of-care diagnostics, which require in situ and long-term measurements, it is important to have sensors that are robust, miniaturized, as well as calibration- and maintenance-free [1,2,3]. Classical ion sensors are typically potentiometric ion-selective electrodes (ISEs) that offer a wide linear range and good detection limits under optimized conditions [4]. Conventional ISEs contain an internal filling solution essential to transform ionic to electronic conductivity, but it is also a major hindrance to achieving robustness and miniaturization [2,3,5]. The discovery of “solid contact” (SC) materials capable of replacing the internal filling solution enabled the fabrication of the so-called solid-contact ion-selective electrodes (SCISEs) [6]. Since then, numerous studies have been made towards developing electrode materials and new operational sensing modes for improved sensitivity, stability, and overall performance [7,8,9].

Despite these advances in potentiometric SCISEs, many challenges remain, such as obtaining stable and reproducible standard potentials, limited sensitivity and the precision of the potentiometric method. In 2015, Bobacka’s group introduced a three-electrode coulometric signal transduction method for SCISEs [10]. In this method, a constant potential is kept between conventional reference electrode and SCISE connected as a working electrode. The change in the primary ion activity alters the boundary potential at the ion-selective membrane/solution interface, which gives rise to the flow of a transient reducing/oxidizing current between the SCISE and the counter electrode until an opposite potential change is established at the solid contact. The total cumulated charge (*Q*), obtained from the integration of the transient current with time, was found to be linearly proportional to the change in the logarithm of activity of the measured ion. Signal amplification and sensitivity can be achieved by fine-tuning different parameters during electrode fabrication such as increasing the electrode area, increasing the redox capacitance of the solid contact, and/or decreasing the ion-selective membrane thickness [11].

Several configurations of this experimental setup have been investigated to provide alternative and versatile readout strategies alongside signal amplification and stabilization. Connecting the indicator electrode (ISE) as reference electrode was originally introduced by Crespo et al. [12] when employing a readout method based on electrogenerated chemiluminescence. Recently, the conversion of potentiometric responses to amperometric and coulometric signal readout modes was also employed by connecting the SCISE as RE [13,14,15]. These studies employed a two-compartment electrochemical setup. One is the “sample” compartment where analyte recognition takes place and the other is the “detection” compartment where an amperometric signal is generated from the oxidation of H_2_O_2_ [15], or a redox couple in solution [13,14]. The two compartments were connected either by a salt bridge [14,15], or Ag/AgCl wire [13]. Such a setup is complicated and difficult to miniaturize.

This work introduces a novel experimental setup for the coulometric signal transduction method. It is a one-compartment three-electrode electrochemical cell composed of a potassium ISE (conventional or SCISE) connected as the RE, a PEDOT:PSS-based electrode with dummy membrane as WE, and a glassy carbon rod as CE. One of the biggest advantages of this setup over previous works mentioned above is its simplicity where only a single compartment is used, thus eliminating the need for a salt bridge. A high concentration of background electrolyte was used to ensure that the potentiometric analyte recognition occurs only at the ISE connected as RE, while current flows between the WE and the CE. The possibility of using different types of ISEs (conventional or SCISEs) makes the setup very versatile in comparison to the original configuration which is limited to SCISEs. The influence of parameters such as the thickness of the dummy membrane and redox capacitance of the PEDOT:PSS film in the WEs were investigated. Analytical performance characteristics such as linearity of *Q* versus the logarithm of analyte activity, sensitivity, and electrode-to-electrode reproducibility were assessed. 

## 2. Experimental

### 2.1. Reagents 

Potassium ionophore I (valinomycin), potassium tetrakis[3,5-bis(trifluoromethyl)phenyl)] borate (KTFPB), tetradodecylammonium tetrakis(4-chlorophenyl) borate (ETH-500), bis(2-ethylhexyl) sebacate (DOS), high molecular weight polyvinyl chloride (PVC), and tetrahydrofuran (THF, ≥99.5%) were Selectophore reagents purchased from Sigma-Aldrich (St. Louis, MO, USA). Poly(sodium 4-styrenesulfonate) (NaPSS, average Mw ∼70,000), 3,4-ethylenedioxythiophene (EDOT, 97%) sodium chloride (NaCl, ≥99.9995%), potassium chloride (KCl, ≥99.999%), and multi-walled carbon nanotubes (MWCNTs) (≥95%, O.D x L: 6–9 nm × 5 µm) were purchased from Sigma-Aldrich (St. Louis, MO, USA). Distilled and deionized water, resistivity 18.2 MΩ cm (ELGA Purelab Ultra, ELGA LabWater, Lane End, United Kingdom) was used to prepare the aqueous solutions.

### 2.2. Electrode Preparation

#### 2.2.1. Preparation of the Plasticized PVC-Based Membranes

Different plasticized PVC-based membranes were prepared. The composition of the potassium ion-selective membrane (K^+^- ISM) in % (*w*/*w*) was: 1.0 valinomycin, 0.5 KTFPB, 1.0 ETH500, 65.0 DOS, and 32.5 PVC. These components were dissolved in THF to obtain cocktails with 15 and 9.76% dry mass for making the solid-state and conventional potassium ion-selective electrodes (K^+^- ISEs), respectively. A PVC-based cocktail for preparing a dummy membrane (DM) was made using all the previously mentioned membrane components except the ionophore valinomycin, and had a final dry mass content of 14.9%. 

#### 2.2.2. Preparation of GC/PEDOT:PSS/K^+^- ISM Electrodes

Glassy carbon (GC) rods (Sigradur HTW, Thierhaupten, Germany) with diameters (*Ø*_GC_) of 3 and 5 mm, mounted in polyvinyl chloride (PVC) cylinders, were used for preparing the electrodes. The GC electrodes were polished with sandpaper with increasing grit (180, 240, 600, 800, 1000, 1200), followed by diamond paste (15, 9, 3, and 1 µm), then with 0.3 µm alumina slurry. Finally, the electrodes were ultra-sonicated for 15 min in ethanol and then in deionized water. 

For the electrochemical synthesis of PEDOT:PSS, a deaerated polymerization solution containing 10^−2^ M of the monomer (EDOT) and 10^−1^ M NaPSS as supporting electrolyte was used. The Autolab general purpose electrochemical system (AUT30.FRA2-Autolab, Eco Chemie, B.V., Utrecht, The Netherlands) was used with a three-electrode electrochemical cell. A double-junction Ag/AgCl/3 M KCl/0.1 M NaPSS (Metrohm, Herisau, Switzerland) and a GC rod were used as the reference and counter electrode, respectively. 

Using the galvanostatic polymerization method, a constant current of 2 µA/mm^2^ was applied between the working and counter electrodes for 142 s to achieve the polymerization charge (*Q*_polym_) of 2 mC. Afterwards, the resulting electrodes, i.e., GC/PEDOT:PSS, were rinsed with deionized water and conditioned in a 10^−2^ M KCl for at least 24 h. The conditioned GC/PEDOT:PSS electrodes were rinsed with deionized water and left for 24 h. A 50 µL portion of the K^+^- ISM cocktail was used to prepare each GC/PEDOT:PSS/K^+^- ISM electrode that was then conditioned in 10^−2^ M KCl solution before further use and in-between measurements.

#### 2.2.3. Preparation of GC/MWCNTs/ K^+^- ISM Electrodes

A MWCNTs dispersion was prepared by adding 1 mL of tetrahydrofuran to 0.005 g of MWCNTs to achieve a final concentration of 0.5 wt%. The mixture was vortexed at 2500 rpm for two minutes and ultra-sonicated for two hours. A 10 µL portion of the MWCNTs dispersion was drop-casted on top of the GC (*Ø*_GC_ = 3 mm) substrate in two portions and the electrodes were left to dry. Subsequently, the GC/MWCNTs electrodes were conditioned overnight in 10^−2^ M KCl solution. After conditioning, the electrodes were left to dry and later 50 µL of the K^+^- ISM cocktail was drop-casted on top of the MWCNTs layer. The resulting GC/MWCNTs/ K^+^- ISM electrodes were conditioned in 10^−2^ M KCl solution before further use and in-between measurements.

#### 2.2.4. Preparation of the Conventional K^+^- ISEs

For making conventional K^+^- ISEs, the membrane cocktail was poured into a glass cylinder with diameter of 24 mm fixed on a glass plate. The membrane was left to dry for ca. 48 h. Once dried, disks of 8 mm diameter were punched from the main membrane and put in the sensor body (Philips 1S561, Möller glasbläserie, Zürich, Switzerland). As inner filling solution, 10^−2^ M KCl was used. The resulted conventional K^+^- ISEs were conditioned in a 10^−2^ M KCl solution before further use and in-between measurements.

#### 2.2.5. Preparation of GC/PEDOT:PSS/DM Electrodes

The electrodes with the dummy membrane, acting as the working electrodes in the studied set-up, were prepared using a GC substrate (*Ø*_GC_ = 3 mm) covered with a layer of PEDOT:PSS (10 mC or 20 mC). A 25 μL portion of the dummy membrane (DM) cocktail was drop casted on top of the GC/PEDOT:PSS layer. In a similar way, 41.7 μL of DM cocktail was drop-casted on the electrodes with bigger GC substrate (*Ø*_GC_ = 5 mm) covered with PEDOT:PSS (27.8 mC or 55.5 mC). The thickness of the PEDOT:PSS layer was the same for the small (*Ø*_GC_ = 3 mm) and big (*Ø*_GC_ = 5 mm) electrodes. Likewise, the thickness of the DM was similar in both small and big electrodes. 

The prepared electrodes were left to dry for ca. 48 h. Afterwards, they were conditioned in 10^−2^ M KCl solution for at least 24 h before further use.

### 2.3. Characterization Methods

#### 2.3.1. Electrochemical Impedance Spectroscopy

Electrochemical impedance spectroscopy measurements were performed in a conventional one-compartment three-electrode electrochemical cell using an Autolab General Purpose Electrochemical System (AUT20.FRA2-Autolab, Eco Chemie, B.V., Utrecht, The Netherlands) and the Autolab Frequency Response Analyzer (FRA) software. The impedance spectra were recorded in 10^−1^ M KCl solution at the open-circuit potential (OCP) using the frequency range of 100 kHz–10 mHz and excitation amplitude of 10 mV. A glassy carbon rod and Ag/AgCl/3 M KCl reference electrode (6.0733.100 Metrohm, Herisau, Switzerland) were used as the counter and reference electrode, respectively. 

#### 2.3.2. Potentiometric Measurements

A 16-channel millivoltmeter (Lawson Labs. Inc., Malvern, PA, USA) and a two-electrode electrochemical cell composed of the K^+^- ISEs and a Metrohm double junction Ag/AgCl/3 M KCl/1 M LiOAc reference electrode was used for the potentiometric measurements carried out using the prepared electrodes (GC/PEDOT:PSS/K^+^- ISM, GC/MWCNTs/K^+^- ISM, GC/PEDOT:PSS/DM, and conventional K^+^- ISE). 

For calibrating the K^+^- ISEs, a 50.0 mL starting solution of 10^−2^ M KCl was used. Sequential dilution was performed automatically using a Metrohm Dosino 700 instrument equipped with two 50 mL burettes (Herisau, Switzerland). The calibration was performed from 10^−2^ to 10^−7^ M KCl. At each concentration, the potential was measured every 0.2 s for five minutes. Calibration with background electrolyte (BGE) was performed using 50.0 mL of 10^−2^ M KCl with 10^−1^ M NaCl as BGE as the starting solution and 10^−1^ M NaCl solution as the diluent. The activity coefficients were calculated using the extended Debye-Hückel equation.

The sensitivity of the GC/PEDOT:PSS electrodes, with and without the dummy membrane, to the presence of O_2_ and CO_2_ gases in the sample was studied in 10^−3^ M KCl solution. 

#### 2.3.3. Chronoamperometric and Chronocoulometric Measurements

The chronoamperometric and chronocoulometric experiments were performed using an IviumCompactStat (Ivium Technologies, Eindhoven, The Netherlands). Each of the K^+^- ISEs (GC/PEDOT:PSS/K^+^- ISM, GC/MWCNTs/K^+^- ISM, or conventional K^+^- ISE) was connected as the reference electrode in the three-electrode electrochemical cell kept in a Faraday cage to reduce the background noise. The GC/PEDOT:PSS/DM and a GC rod were connected as working and counter electrode, respectively.

Prior to the actual experiment, a chronopotentiometric measurement was taken for 15 min with constant stirring to determine the OCP of the electrode. Current baseline correction was performed. 

Sequential dilution was performed automatically using a Metrohm Dosino 700 instrument equipped with two 50 mL burettes (Herisau, Switzerland).

## 3. Results and Discussions

### 3.1. Electrochemical Impedance Spectroscopy Measurements

**Figure 1:**
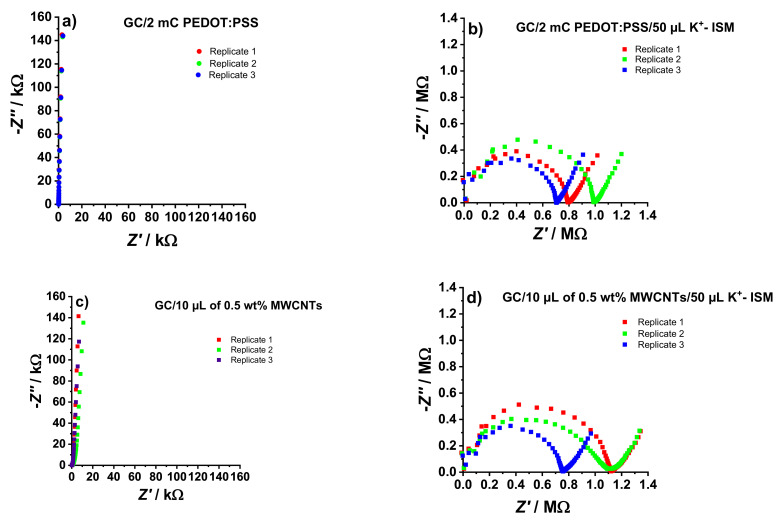
Electrochemical impedance spectra of PEDOT:PSS-based (**a**,**b**) and MWCNTs-based (**c**,**d**) electrodes recorded in 10^−1^ M KCl at OCP, in the frequency range of 100 kHz–10 mHz, and excitation amplitude of 10 mV.

The impedance spectra of the K^+^-SCISEs were measured before and after drop-casting the K^+^- ISM. As shown in Figure 1, a close to vertical line can be seen in the impedance spectra of both GC/PEDOT:PSS (Figure 1a) and GC/MWCNTs (Figure 1c). This is typical for PEDOT:PSS [16] and MWCNTs [17] electrodes indicating a capacitive mechanism related to the bulk redox capacitance and double-layer capacitance of the PEDOT:PSS and MWCNTs films, respectively.

While the 90° capacitive line in the GC/PEDOT:PSS electrodes (Figure 1a) extends to the low-frequency region of the spectra, a slight deviation is noticeable for the GC/MWCNTs (Figure 1c). This indicates that there is a small diffusion contribution in the transduction process of MWCNTs, which can be attributed to the inhomogeneous quality of the MWCNTs film due to manual deposition [17]. Moreover, good repeatability was observed in the impedance spectra of the electrodes with galvanostatically polymerized PEDOT:PSS (Figure 1a), compared to the manually added MWCNTs film (Figure 1c).

The low-frequency capacitance (*C*_LF_) of GC/PEDOT:PSS and GC/MWCNTs electrodes were estimated using Equation (1) below, where *f* is the lowest frequency value used in the EIS measurement (0.01 Hz) and -*Z*″ is the corresponding imaginary impedance.

*C*_LF_ = −1/(2π*fZ*″) (1)

The obtained *C*_LF_ values for GC/PEDOT:PSS and GC/MWCNTs were 110.6 ± 0.7 µF and 122.0 ± 12.2 µF, respectively, confirming the excellent repeatability of the GC/PEDOT:PSS electrodes.

Compared to the spectra shown in Figure 1a,c, which are dominated by a capacitive line, the spectra in Figure 1b,d are dominated by a high-frequency semicircle. This is related to the geometric capacitance and the bulk resistance of the ISM (*R*_ISM_), determined by estimating the diameter of the semicircle [11,18]. The poor repeatability of the calculated *R*_ISM_ for the prepared K^+^-SCISEs is caused by the manual drop-casting of the K^+^- ISM. The *R*_ISM_ values from the EIS measurements are summarized in Table 1.

A low-frequency line with an angle of ca. 45° (Warburg impedance) was seen in the impedance spectra of the electrodes after adding the K^+^- ISM, i.e., GC/PEDOT:PSS/K^+^- ISM (Figure 1b) and GC/MWCNTs/K^+^- ISM (Figure 1d). This corresponds to the diffusion-limited ion-to-electron transduction mechanism in the electrodes. Estimating the low-frequency capacitance (*C*_LF_) from −*Z*″ at the lowest frequency by using Equation (1) gives the values summarized in Table 1. The *C*_LF_ values are significantly lower after the PEDOT:PSS and MWCNTs are coated with the K^+^- ISM, in good agreement with earlier observations [19].

**Figure 2:**
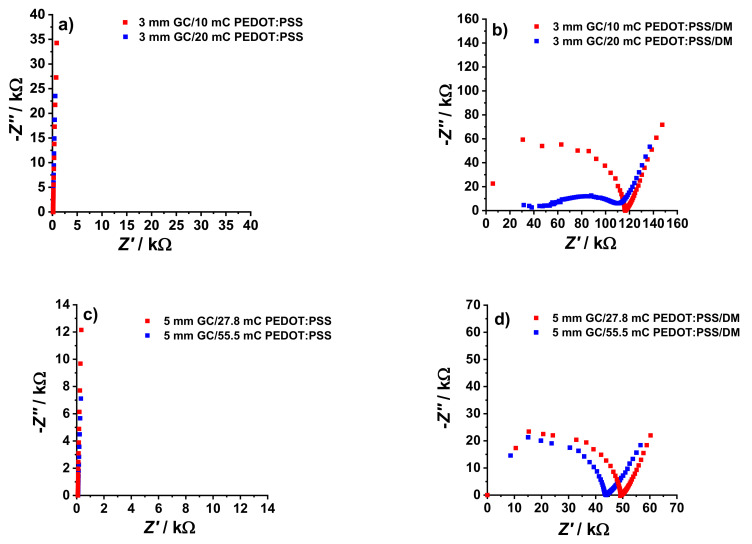
Electrochemical impedance spectra of GC/PEDOT:PSS electrodes with *Ø*_GC_ = 3 mm (**a**,**b**), *Ø*_GC_ = 5 mm (**c**,**d**), and different *Q*_polym_ (as indicated in the figures) recorded in 10^−1^ M KCl at OCP in the frequency range of 100 kHz–10 mHz and excitation amplitude of 10 mV.

The impedance spectra of the GC/PEDOT:PSS electrodes before and after the deposition of the DM are shown in Figure 2. The spectra for GC/PEDOT:PSS are dominated by the 90° capacitive line (Figure 2a,c), while a high-frequency semicircle dominates after the deposition of the dummy membrane (Figure 2b,d). 

The *C*_LF_ and *R*_DM_ values shown in Table 2 were estimated in the same way as for the K^+^-SCISEs. The redox capacitance of the PEDOT:PSS film was varied in two ways: one is by increasing the film thickness and another is by using electrodes with larger *Ø*_GC_, and corresponding area (*A*_GC_). Ideally, the redox capacitance of the film is directly proportional to *Q*_polym_ [16] which was generally observed in the results of the EIS experiments. As shown from the results in Table 2, doubling the *Q*_polym_ (and thus the PEDOT:PSS film thickness) at constant *Ø*_GC_ and DM thickness resulted in a less than two times (ca. 1.4 times) increase in *C*_LF_. Interestingly, ca. 2.8 times increase in electrode area (*A*_GC_) while keeping the thickness of the PEDOT:PSS film and the dummy membrane constant, resulted in ca. 2.8 times increase in *C*_LF_. This shows that there is a better correlation between *A*_GC_ and *C*_LF_ compared to the correlation between *Q*_polym_ and *C*_LF_. 

It has been proven in previous studies [18] that increasing the electrode area with constant thickness of the CP film and low membrane resistance increases the accessibility of the redox capacitance of the solid-contact layer, and that is why the ratio between the change in *C*_LF_ and in *A*_GC_ is close to one. A different behavior is seen when the thickness of the PEDOT:PSS film is increased at constant *A*_GC_. The redox capacitance of thicker CP films becomes less accessible, so it is difficult to fully utilize the material as ion-to-electron transducer. This could be the reason why doubling the film thickness only resulted in only ca. 1.4 times the *C*_LF_ value. Lastly, since the results from the impedance spectra is dominated by the high-frequency semicircle, the *C*_LF_ did not have much effect on the time constant *τ* compared to *R*_DM_. The time constant, *τ*, which is the product of *R*_DM_ and *C*_LF_, falls within a relatively narrow range (*τ* = 22–31 s) for all the GC/PEDOT:PSS/DM electrodes presented in Table 2. 

### 3.2. Potentiometric Measurements

The results from the calibrations of K^+^- ISEs in KCl solution, with and without 10^−1^ M NaCl as BGE, are shown in Table 3. In both cases, the electrodes showed similar and close to Nernstian behavior. Except for the conventional K^+^- ISEs, which are known to have stable standard potential (*E*°), a large variation in the standard potential was observed for the other studied electrodes. 

The use of a single electrochemical cell in the proposed novel experimental setup was made possible by the presence of a constant BGE in the sample solution. This eliminated the need for having separate sample and detection cells connected by a salt bridge, which is an advantage over previous studies employing the ISE as the RE [13,14,15]. However, it is important to ensure that the analyte recognition takes place only at the K^+^- ISE. 

**Figure 3:**
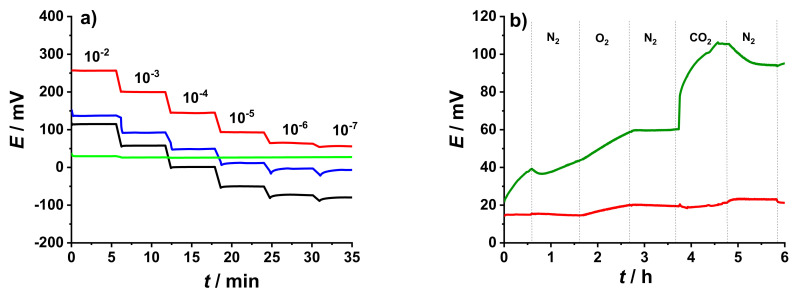
(**a**) Comparison between the potentiometric response of different K^+^- ISEs: GC/PEDOT:PSS/50 µL K^+^- ISM (black line), GC/MWCNTs/50 µL K^+^- ISM (red line), and conventional K^+^- ISE (blue line), and GC/10 mC PEDOT:PSS/25 μL DM (green line) in 10^−2^ M to 10^−7^ M KCl solution with 10^−1^ M NaCl as BGE. (**b**) Gas sensitivity of electrodes: GC/10 mC PEDOT:PSS (green line) and GC/10 mC PEDOT:PSS/25 μL DM (red line) carried out in 10^−3^ M KCl solution.

To highlight the difference in response of K^+^- ISEs and GC/PEDOT:PSS/DM electrodes to changes in K^+^ ion concentration, the potential of these electrodes was measured in the presence of 10^−1^ M NaCl as BGE (Figure 3a). As expected, the presence of a BGE does not affect the performance of the K^+^- ISEs. On the other hand, the GC/PEDOT:PSS/DM electrodes was not sensitive to the change in K^+^ ion concertation due to the high Na^+^ concentration in the BGE.

Earlier studies revealed that conducting polymer films used as solid-contacts have shown sensitivity to gases such as O_2_ and CO_2_ [20,21]. Figure 3b shows results from the gas sensitivity measurement conducted in 10^−3^ M KCl solution using GC/PEDOT:PSS electrodes with and without the DM. Introducing O_2_ gas in the solution resulted in ca. 10 mV increase in potential of the GC/PEDOT:PSS electrodes. The presence of CO_2_ gas in the solution has the most distinct effect on the potential readings (ca. 50 mV) due to the accompanying pH change [20] as a consequence of the dissolution of CO_2_ gas in the solution. As can be seen in Figure 3b, adding the DM almost eliminated the sensitivity of the electrode to the presence of dissolved gases and pH changes in the sample. 

### 3.3. Chronoamperometric and Chronocoulometric Measurements

In the original setup for constant-potential coulometry a SCISE was connected as the WE, a glassy carbon rod and a conventional Ag/AgCl/3M KCl were used as the counter and reference electrode, respectively [10,22].

Recently, a modified set-up was prosed using a two-compartment electrochemical cell. In one compartment containing a redox couple, the counter and working electrodes were placed. In the other compartment, a SCISE was placed and connected as reference electrode. The two compartments were connected by a salt-bridge [14,15] or Ag/AgCl wire [13]. In this work, a single-cell approach is presented which is simple and easy to miniaturize. The ISE was connected as the reference electrode, the glassy carbon rod as the counter electrode, and GC/PEDOT:PSS/DM as the working electrode. The advantage of using the GC/PEDOT:PSS/DM is the easy control and adjustment of the electrode capacitance and resistance which influence the time constant and thus the response time of the electrode. In addition, the presence of the dummy membrane almost eliminates the sensitivity of the electrode to the presence of dissolved gases and pH changes in the sample. 

**Figure 4:**
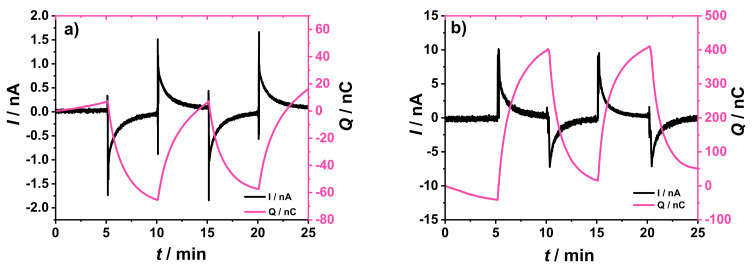
Chronoamperometric and chronocoulometric response collected using (**a**) the original setup (WE: GC/2 mC PEDOT:PSS/50 µL K^+^-ISM, RE: Ag/AgCl/3 M KCl/1 M LiOAc, CE: GC rod), and (**b**) the novel experimental setup (WE: GC/10 mC PEDOT:PSS/25 µL DM, RE: GC/2 mC PEDOT:PSS/50 µL K^+^-ISM, CE: GC rod) for the coulometric signal transduction. The addition/dilution steps correspond to a 5% increase/decrease of K^+^ concentration in a starting solution of 10^−3^ M KCl + 10^−1^ M NaCl as BGE.

The comparison between the chronoamperometric and chronocoulometric plots obtained using the original and the novel experimental setups is shown in Figure 4. The GC/2 mC PEDOT:PSS/50 µL K^+^- ISM electrode was connected as the WE with a double junction Ag/AgCl/3 M KCl/1 M LiOAc as the RE in the original setup (Figure 4a). Alternatively, the K^+^- ISE was connected as the RE in the new setup and a GC/10 mC PEDOT:PSS/25 µL DM electrode was connected as the WE (Figure 4b). In both cases a GC rod was used as CE.

It should be noted that the direction of the current changes is opposite in these two cases because the ISE was connected as working electrode in the original setup (Figure 4a) and as reference electrode in our new setup (Figure 4b).

As shown in Figure 4a,b, both setups showed good reversibility and signal repeatability after two consecutive addition/dilution steps. The new setup (Figure 4b) shows an improvement (at least ca. five times increase) of the current and charge signals, without sacrificing the equilibration time, when compared with the original setup (Figure 4a). For both setups there is a drift in the charge curve (0–5 min), which must be related to minor potential drifts in either WE or RE (or both) that results in a small bias current in the chronoamperometric curve and, hence, a drift in the charge. 

Similar results were obtained using other ISEs, as reference electrode, such as the GC/10 µL of 0.5 wt% MWCNTs/50 µL K^+^- ISM and conventional K^+^- ISE (results not shown). The results are independent of the type of the ISE used as reference electrode as long as it has a Nernstian behavior. 

#### 3.3.1. Effect of Increasing the Thickness of the Dummy Membrane in the WEs

**Figure 5:**
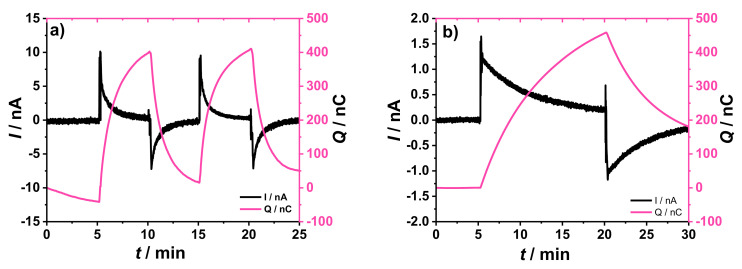
Chronoamperometric and chronocoulometric response in the addition/dilution experiments using working electrodes with different DM thicknesses: (**a**) 3 mm GC/10 mC PEDOT:PSS/25 µL DM and (**b**) 3 mm GC/10 mC PEDOT:PSS/100 µL DM. The addition/dilution steps correspond to a 5% increase/decrease of K^+^ concentration in a starting solution of 10^−3^ M KCl + 10^−1^ M NaCl as BGE.

The effect of increasing the thickness of dummy membrane in the WE was studied using electrodes with similar *Ø*_GC_ and PEDOT:PSS film loading but with different DM thicknesses. As shown in Figure 5, electrodes with thicker DM resulted in current peaks with smaller amplitude and longer equilibration time greater than 15 min, as expected. Consequently, only one addition/dilution step was performed for the working electrodes with thicker dummy membranes.

It has been previously revealed through theoretical modelling that the peak current is inversely affected by the cell resistance [23]. From the results of the EIS measurements, it was confirmed that the addition of the dummy membrane provided a certain resistance *R*_DM_ to the electrode connected as WE. Moreover, *R*_DM_ influences the time constant (*τ*). In addition to the capacitance and resistance, mass transport across the membrane could also affect the response time, which explains why thinner membranes are typically preferable [11,23]

The results show that the cumulated charge Q is independent of the DM thickness as Q is the integration of the current with time [11,23]. Although reversible, it can be seen in Figure 5b that the current did not return to the baseline and the residual current affected the magnitude of the reverse charge peak. The same results were obtained using other ISEs as reference electrode. For this reason, the rest of the experiments were performed only with the working electrodes having a thinner DM.

#### 3.3.2. Effect of Increasing the Redox Capacitance of the PEDOT:PSS Film in the WEs

The influence of the redox capacitance of the PEDOT:PSS film in GC/PEDOT:PSS/DM electrode on the signal amplification and equilibration time was investigated. Working electrodes with equivalently thin dummy membrane but with different *Ø*_GC_ and *Q*_polym_ were examined in the addition/dilution experiments with Δ*C*_K+_ = 5%. In general, increasing the *Q*_polym_ (and consequently the redox capacitance of PEDOT:PSS film) at constant *Ø*_GC_ resulted in signal amplification. From the EIS experiments, it was observed that the time constant τ is also influenced by the redox capacitance of the conducting polymer films although not as prominently as the effect of *R*_DM_. Increasing the amount of PEDOT:PSS film means that more material participates in the redox reaction to achieve the desired ∆*E* [22,23], resulting in longer equilibration time.

**Figure 6:**
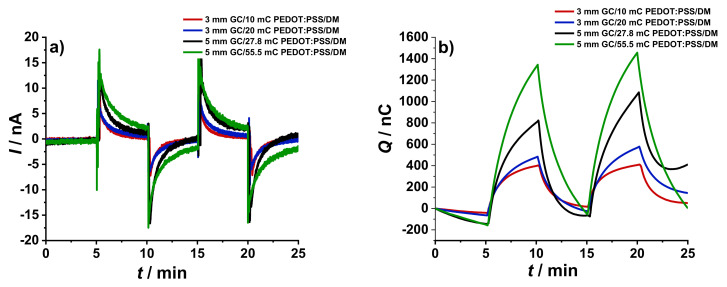
Chronoamperometric (**a**) and chronocoulometric (**b**) response during the addition/dilution experiments using different PEDOT:PSS-based WEs with equivalently thin dummy membrane and a GC/2 mC PEDOT:PSS/50 µL K^+^-ISM connected as the RE and GC rod as the CE. The addition/dilution steps correspond to a 5% increase/decrease in K^+^ concentration in a starting solution of 10^−3^ M KCl + 10^−1^ M NaCl as BGE.

From the experimental results shown in Figure 6, it is notable that increasing the thickness of the PEDOT:PSS layer in WEs with the same *Ø*_GC_ and DM thickness did not influence the amplitude of the current peak. Instead, it resulted in peak broadening and longer equilibration time, which has been consistently observed in both theoretical and experimental studies [11,18,23]. Increasing the *Ø*_GC_ from 3 mm to 5 mm with constant PEDOT:PSS film thickness with *Q*_polym_ 10 mC and 27.8 mC, respectively seemed to be a better approach. Increasing the redox capacitance accompanied by larger electrode area, compared to just making the CP film thicker, allowed for better access to the electroactive material. This resulted in higher and sharper current peaks without any significant increase in the response time (Figure 6a). 

Integration of the current with time resulted in a more pronounced relationship between the total cumulated charge *Q* and the redox capacitance of the PEDOT:PSS film. At constant DM thickness, it is expected that at higher sensitivity is achieved with increased redox capacitance, accompanied by a longer equilibration time [11]. As shown in Figure 6b, the change in *Q* values after each addition/dilution step increases with increasing the redox capacitance of the PEDOT:PSS film. Moreover, the electrode with lowest *Q*_polym_ (3 mm GC/10 mC PEDOT:PSS/DM), showed the shortest response time, indicated by the appearance of a plateau-like region in the graph. Indeed, it is important to find a compromise between the amplitude of the current peak, response time and *Q* to achieve useful results. The same results and interpretations were obtained for the setups with GC/10 µL 0.5 wt% MWCNTs/50 µL K^+^- ISM and conventional K^+^- ISE connected as the RE (results not shown).

#### 3.3.3. Calibration Experiment

**Figure 7:**
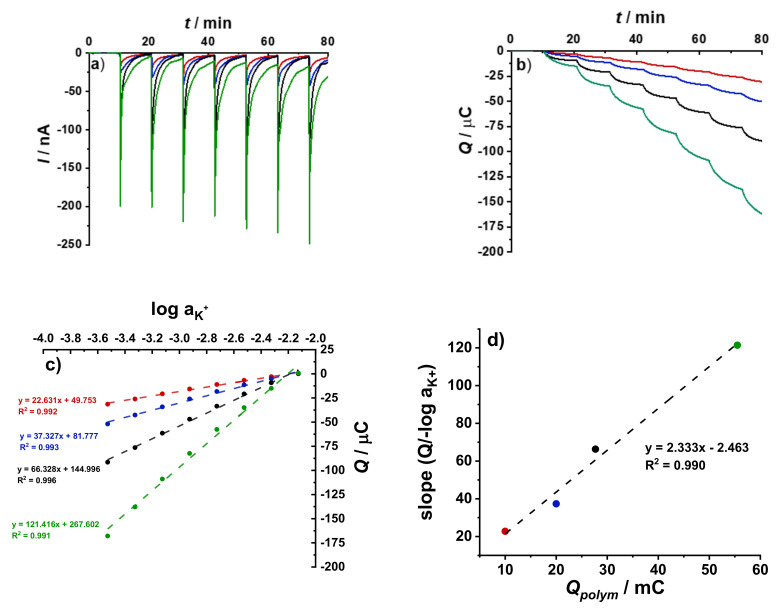
Chronoamperometric (**a**), chronocoulometric (**b**) response, cumulated charge *Q* vs. loga_K+_ (**c**) during the calibration conducted in 10^−2^ M to 10^−3.4^ M KCl + 10^−1^ M NaCl as BGE with ∆log*C*_K+_ = 0.2 decade step^−1^; 3 mm GC/10 mC PEDOT:PSS/DM (red), 3 mm GC/20 mC PEDOT:PSS/DM (blue), 5 mm GC/27.8 mC PEDOT:PSS/DM (black), or 5 mm GC/55.5 mC PEDOT:PSS/DM (green) with equivalent dummy membrane thickness were used as WEs. GC/2 mC PEDOT:PSS/50 µL K^+^- ISM and GC rod were connected as the RE and CE, respectively. Slope values from results in (**c**) against the polymerization charge *Q*_polym_ of the PEDOT:PSS film in the WE (**d**).

Based on the observed response times, electrodes with thin DM were used in this experiment. The electrochemical cell consisted of GC/2 mC PEDOT:PSS/50 µL K^+^- ISM electrode connected as the RE, a GC rod connected as the CE. As the WEs, GC/PEDOT:PSS/DM electrodes with *Ø*_GC_ = 3 and 5 mm, and various *Q*_polym_ were used. The results obtained from the successive dilution performed from 10^−2^ M to 10^−3.4^ M KCl solutions with 10^−1^ M NaCl as BGE with ∆ log*C*_K+_ = 0.2 decade/ step are shown in Figure 7. 

The results show that increasing the redox capacitance at constant *Ø*_GC_ and DM thickness resulted in broader peaks and longer response times. Because of the larger dilution steps, the measurement time was extended from 5 to 10 min for each concentration step. Figure 7a shows that the current peaks are quite reproducible throughout all the dilution steps. The drifting current baseline, most pronounced in the large electrode with the thicker PEDOT:PSS film and highest redox capacitance, is due to the insufficient time for equilibration in between the dilution steps. 

The coulometric response and the resulting calibration curves in Figure 7b,c, respectively, show good linear relationship between the *Q* and log *a*_K_^+^ values. As shown in Figure 7d, there is a linear relationship between *Q*_polym_ and the slopes from the calibration curves in Figure 7c. However, the curve is not perfectly linear, due to the difference in *Ø*_GC_ of the electrodes. Doubling the PEDOT:PSS film thickness resulted in ca. 1.7 times increase in the slope value. Moreover, enlargement of *Ø*_GC_ by ca. 2.8 times, at constant PEDOT:PSS film thickness, resulted in ca. 3.1 times increase in slope value. This supports the results from EIS measurements, that spreading out the conducting polymer film over a larger area gives better accessibility to its redox capacitance as compared to depositing a thicker film on the same electrode area.

**Figure 8:**
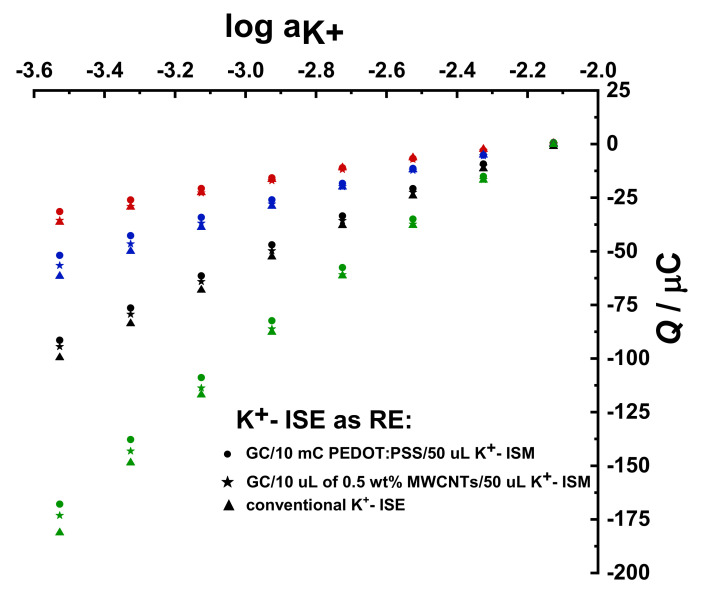
Cumulated charge *Q* vs. log *a*_K+_ from the calibration conducted in 10^−2^ M to 10^−3.4^ M KCl + 10^−1^ M NaCl as BGE with ∆log*C*_K+_ = 0.2 decade step^−1^; 3 mm GC/10 mC PEDOT:PSS/DM (red), 3 mm GC/20 mC PEDOT:PSS/DM (blue), 5 mm GC/27.8 mC PEDOT:PSS/DM (black), and 5 mm GC/55.5 mC PEDOT:PSS/DM (green) with equivalent dummy membrane thickness were used as WEs. Different K^+^- ISEs were used as REs, as indicated in the figure.

Similar results were obtained using a GC/10 µL 0.5 wt% MWCNTs/50 µL K^+^- ISM WE and a conventional K^+^- ISE connected as the RE (results not shown). Figure 8 shows that the obtained results are independent of the type of K^+^- ISE, whether all-solid-state or conventional, used as RE. This means that the novel experimental setup can be assembled using different electrode combinations to suit the demands of an experiment.

#### 3.3.4. Detection of Small Concentration Changes

**Figure 9:**
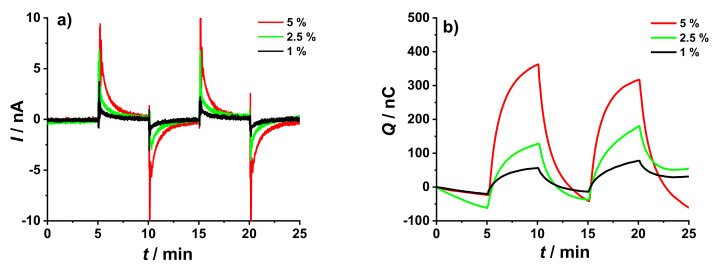
Chronoamperometric (**a**) and chronocoulometric (**b**) response resulted from the addition/dilution experiments using a 3 mm GC/10 mC PEDOT:PSS/25 µL DM as the WE, GC/2 mC PEDOT:PSS/50 µL K^+^- ISM as RE, and GC rod as CE. The addition/dilution steps correspond to 5%, 2.5%, and 1% increase/decrease in K^+^ concentration conducted in a starting solution of 10^−3^ M KCl + 10^−1^ M NaCl as BGE.

The electrode responses in the new setup were further investigated using addition/dilution experiments with Δ*C*_K+_ = 5%, 2.5%, and 1%, in the presence of 0.1 M NaCl as BGE. Figure 9 shows results obtained using the 3 mm GC/10 mC PEDOT:PSS/25 µL DM electrode as the WE and GC/2 mC PEDOT:PSS/50 µL K^+^- ISM as the RE. As expected, smaller changes in K^+^ concentration resulted in smaller current peaks that decay faster as shown in Figure 9a. Naturally, a larger concentration change results in a larger coulometric response, as shown in Figure 9b.

As seen in Figure 9a, a 1% change in concentration resulted in signals with magnitude that are comparable to the 5% change in concentration using the original setup (Figure 4a). This is in good agreement with the fact that the novel experimental setup produced signals that are at least five times larger than the ones resulted from the original setup, as mentioned earlier in Section 3.3. 

## 4. Conclusion

In this work, a novel experimental setup for the coulometric signal transduction method in ion-selective electrodes was studied. A single-compartment electrochemical cell was used, where a K^+^- ISE was connected as the RE, a GC/PEDOT:PSS/DM was connected as WE, and a GC rod was connected as CE. Three different types of K^+^- ISEs were evaluated as the Res: one conventional and two SCISEs with either PEDOT:PSS or MWCNTs as solid contact. The Wes were prepared using GC substrates of two different sizes covered with various thicknesses of the PEDOT:PSS film and dummy membrane. 

The approach employed in this work made it possible to combine the two-compartment sample and detection cells from previous studies into a simple and convenient single-compartment cell. The PEDOT:PSS film in the WE exhibits a cationic response, but the use of a high concentration of BGE effectively masked the sensitivity of the WE to changes in K^+^ concentration. In this case, the potentiometric detection takes place at the K^+^- ISE and the signal transformation occurs at the WE. Furthermore, a layer of PVC-base dummy membrane (without ionophore) was added on top of PEDOT:PSS in the WE to regulate the signal and measurement time. Additionally, potentiometric tests revealed that the dummy membrane made the working electrode insensitive to the presence of gases such as O_2_ or CO_2_ (pH changes) in the sample solution.

The results from the chronoamperometric and chronocoulometric addition/dilution experiments showed that the new setup produced results with improved current and charge signals as compared to the original setup used earlier. Good reversibility and measurement repeatability were exhibited by the current and cumulated charge signals. Using different types of K^+^- ISEs employed as REs, proved that the novel setup is compatible with both solid-contact and conventional ISEs. This demonstrates the versatility of the novel setup compared to the original setup, which is limited to SCISEs.

Increasing the thickness of the dummy membrane in the WE resulted in longer equilibration time. Increasing the redox capacitance of the conducting polymer in the WE produced larger signals at the expense of longer equilibration time. It was observed that in order to increase the redox capacitance of the CP film in the WE, it is favorable to maintain the same film thickness and increase the electrode area rather than maintaining the same electrode area and increasing the thickness of the film.

The calibration curves obtained during sequential dilution experiments showed a good linear relationship between the resulted charge (*Q*) and logarithm of potassium ion activity (log*a*_K+_). Moreover, the determination of smaller changes in the analyte concentration (5%, 2.5%, and 1%) was successful. The new setup using a single compartment eliminates the need for the salt bridge. It is a simple and versatile alternative for coulometric signal transduction compatible with different types of ISEs.

## Figures and Tables

**Table 1:** The low-frequency capacitance (*C*_LF_) and bulk resistance (*R*_ISM_) values calculated from the EIS spectra of K^+^-SCISEs. *S*_d_ = standard deviation (n = 3).

*Ø*_GC_/mm	Solid-Contact Layer	*V*_ISM_/μL	*C*_LF_ ± *S*_d_/μF	*R*_ISM_ ± *S*_d_/kΩ
3	2 mC PEDOT:PSS	50	43.7 ± 0.6	820 ± 142
3	10 µL 0.5 wt% MWCNTs	50	52.1 ± 2.0	998 ± 206

**Table 2:** The low-frequency capacitance (*C*_LF_), bulk resistance (*R*_DM_) and time constant (*τ*) values calculated from the EIS spectra of the fabricated GC/PEDOT:PSS/DM electrodes.

*Ø*_GC_/mm	*A*_GC_/mm^2^	*I*_polym_/μA	*t*_polym_/s	*Q*_polym_/mC	*V*_DM_/μL	*C*_LF_/μF	*R*_DM_/kΩ	*τ*/s
3	7.07	14.14	707.2	10	25	207.9	111.4	23.2
			1414.2	20	25	298.4	78.5	23.4
5	19.6	39.26	707.2	27.8	41.7	574.9	39.1	22.5
			1414.2	55.5	41.7	864.6	35.3	30.5

*Ø*_GC_ = diameter of the GC substrate, *A*_GC_ = area of the GC substrate, *I*_polym_ = polymerization current, *t*_polym_ = polymerization time, *Q*_polym_ = polymerization charge, *V*_DM_ = volume of the dummy membrane cocktail.

**Table 3:** Results from the potentiometric measurements of K^+^- SCISEs. *S*_d_ = standard deviation (n = 3).

K^+^- SCISE	Slope ± *S*_d_/mV Decade^−1^		*E*°± *S*_d_ /mV	
	in KCl	in KCl + 10^−1^ M NaCl	in KCl	in KCl + 10^−1^ M NaCl
GC/2 mC PEDOT:PSS /50 µL K^+^- ISM	56.8 ± 0.3	55.5 ± 0.4	216 ± 31	213 ± 30
GC/10 uL of 0.5 wt% MWCNTs/50 µL K^+^- ISM	54.8 ± 1.9	53.7 ± 1.6	258 ± 61	253 ± 62
Conventional K^+^- ISE	55.5 ± 0.1	55.2 ± 0.2	256 ± 0.7	254 ± 0.6

## Data Availability

The data presented in this study are available on request from the corresponding author.

## References

[1] Hanr ahan G., Patil D.G., Wang J. (2004). Electrochemical sensors for environmental monitoring: Design, development and applications. J. Environ. Monit..

[2] Rousseau C.R., Bühlmann P. (2021). Calibration-free potentiometric sensing with solid-contact ion-selective electrodes. TrAC Trends Anal. Chem..

[3] Van de Velde L., d’Angremont E., Olthuis W. (2016). Solid contact potassium selective electrodes for biomedical applications—A review. Talanta.

[4] Bobacka J., Ivaska A., Lewenstam A. (2008). Potentiometric Ion Sensors. Chem. Rev..

[5] Shao Y., Ying Y., Ping J. (2020). Recent advances in solid-contact ion-selective electrodes: Func-tional materials, transduction mechanisms, and development trends. Chem. Soc. Rev..

[6] Cadogan A., Gao Z., Lewenstam A., Ivaska A., Diamond D. (1992). All-solid-state sodium-selective electrode based on a calixarene ionophore in a poly(vinyl chloride) membrane with a polypyrrole solid contact. Anal. Chem..

[7] Huang M.-R., Gu G.-L., Ding Y.-B., Fu X.-T., Li R.-G. (2012). Advanced Solid-Contact Ion Selective Electrode Based on Electrically Conducting Polymers. Chin. J. Anal. Chem..

[8] Jaworska E., Pawłowski P., Michalska A., Maksymiuk K. (2019). Advantages of Amperometric Readout Mode of Ion-selective Electrodes under Potentiostatic Conditions. Electroanalysis.

[9] Kałuża D., Michalska A., Maksymiuk K. (2022). Solid-Contact Ion-Selective Electrodes Paving the Way for Improved Non-Zero Current Sensors: A Minireview. ChemElectroChem.

[10] Hupa E., Vanamo U., Bobacka J. (2015). Novel Ion-to-Electron Transduction Principle for Solid-Contact ISEs. Electroanalysis.

[11] Han T., Mattinen U., Bobacka J. (2019). Improving the Sensitivity of Solid-Contact Ion-Selective Electrodes by Using Coulometric Signal Transduction. ACS Sens..

[12] Crespo G.A., Mistlberger G., Bakker E. (2012). Electrogenerated chemiluminescence for potentiometric sensors. J. Am. Chem. Soc..

[13] Han T., Song T., Bao Y., Sun Z., Ma Y., He Y., Gan S., Jiang D., Han D., Bobacka J. (2022). Amperometric response of solid-contact ion-selective electrodes utilizing a two-compartment cell and a redox couple in solution. J. Electroanal. Chem..

[14] Sun X., Yin T., Zhang Z., Qin W. (2022). Redox probe-based amperometric sensing for solid-contact ion-selective electrodes. Talanta.

[15] Yin T., Wang H., Li J., Yuan B., Qin W. (2021). Translating potentiometric detection into non-enzymatic amperometric measurement of H2O2. Talanta.

[16] Bobacka J., Lewenstam A., Ivaska A. (2000). Electrochemical impedance spectroscopy of oxidized poly(3,4-ethylenedioxythiophene) film electrodes in aqueous solutions. J. Electroanal. Chem..

[17] Yuan D., Anthis A.H.C., Ghahraman Afshar M., Pankratova N., Cuartero M., Crespo G.A., Bakker E. (2015). All-Solid-State Potentiometric Sensors with a Multiwalled Carbon Nanotube Inner Transducing Layer for Anion Detection in Environmental Samples. Anal. Chem..

[18] Han T., Vanamo U., Bobacka J. (2016). Influence of Electrode Geometry on the Response of Solid-Contact Ion-Selective Electrodes when Utilizing a New Coulometric Signal Readout Method. ChemElectroChem.

[19] Bobacka J. (1999). Potential stability of all-solid-state ion-selective electrodes using conducting polymers as ion-to-electron transducers. Anal. Chem..

[20] Vázquez M., Bobacka J., Ivaska A., Lewenstam A. (2002). Influence of oxygen and carbon dioxide on the electrochemical stability of poly(3,4-ethylenedioxythiophene) used as ion-to-electron transducer in all-solid-state ion-selective electrodes. Sens. Actuators B Chem..

[21] Vázquez M., Danielsson P., Bobacka J., Lewenstam A., Ivaska A. (2004). Solution-cast films of poly(3,4-ethylenedioxythiophene) as ion-to-electron transducers in all-solid-state ion-selective electrodes. Sens. Actuators B Chem..

[22] Vanamo U., Hupa E., Yrjänä V., Bobacka J. (2016). New Signal Readout Principle for Solid-Contact Ion-Selective Electrodes. Anal. Chem..

[23] Jarolímová Z., Han T., Mattinen U., Bobacka J., Bakker E. (2018). Capacitive Model for Coulometric Readout of Ion-Selective Electrodes. Anal. Chem..

